# Michaelis-Menten Kinetics Measurements of Aldo-Keto Reductases for Various Substrates in Murine Tissue

**DOI:** 10.1016/j.xpro.2020.100206

**Published:** 2020-12-07

**Authors:** Jakob Morgenstern, Elisabeth Kliemank, Marta Campos Campos, Peter Nawroth, Thomas Fleming

**Affiliations:** 1 Department of Internal Medicine I and Clinical Chemistry, University Hospital Heidelberg, Heidelberg 69120, Germany; 2 German Center for Diabetes Research (DZD), Neuherberg 85764, Germany

**Keywords:** Metabolism, Molecular Biology

## Abstract

Aldo-keto reductases (AKRs) are responsible for the detoxification of harmful aldehydes. Due to the large number of isotypes, the physiological relevance of AKRs cannot be obtained using mRNA or protein quantification, but only through the use of enzymatic assays to demonstrate functionality. Here, we present a fast and simple protocol to determine the important Michaelis-Menten kinetics of AKRs, which includes various aldehyde substrates of interest such as 4-hydroxynonenal, methylglyoxal, and malondialdehyde.

For complete details on the use and execution of this protocol, please refer to Morgenstern et al. (2017) and Schumacher et al. (2018).

## Highlights

• Mild and efficient extraction/lysis of mouse tissue
• Monitoring of aldo-keto reductase catalyzed reactions for various substrates
• Comprehensive explanations in order to determine Michaelis-Menten kinetics

## Graphical Abstract

**Figure:**
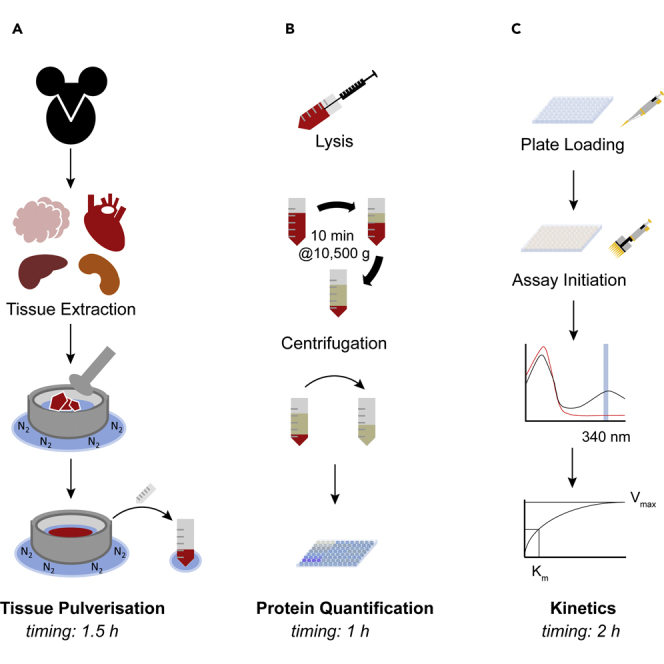

## Before You Begin

This protocol is a straightforward method to determine enzyme kinetics of AKR converted aldehydes in tissue lysates within 4 h. Tissues have to be obtained from mice after vascular perfusion with 1× PBS through the heart and the samples should be snap-frozen after extraction. For tissue processing/crushing, a mortar and pestle needs to be precooled for at least 4 h in a −80°C freezer. Liquid nitrogen is required in order to cool down the materials during the processing. In addition, the lysis buffer should be precooled at 4°C or on ice during the processing. A 1 mL syringe and 20-gauge needle should be prepared for each individual sample and centrifuge rotors should to be precooled at 4°C. For the enzyme assay it is recommended to use a microplate reader but in theory the assay can be run in cuvettes as well using a thermostatic spectrophotometer. Instruments should be kept at 37°C during the assay.

### Cytosolic Lysis Buffer

**Timing: 15 min**

1. Preparation of 50 mL cytosolic lysate buffer (Table 1)
  a) 0.5 mL HEPES of a 1 M stock solution (final concentration 10 mM)
  b) 75 μL MgCl_2_ of a 1 M stock solution (final concentration 1.5 mM)
  c) 0.5 mL KCl of a 1 M stock solution (final concentration 10 mM)
  d) 50 μL EDTA of a 1 M stock solution (final concentration 1 mM)
  e) 25 μL of NP-40 (alternatively Triton X-100) using a syringe to avoid pipette inaccuracy due to viscosity (final concentration, 0.05% v/v)
  f) Adjust to pH 7.4
  g) Buffer can be stored at 4°C for 3 months

**Table 1:** Recipe of Cytosolic Lysis Buffer

Reagent	Final Concentration (mM)	Amount
HEPES (1 M)	10	0.5 mL
MgCl_2_ (1 M)	1.5	75 μL
KCl (1 M)	10	0.5 mL
EDTA (1 M)	1	50 μL
NP-40 or Triton X-100	0.05% (v/v)	25 μL
DTT (1 M)	0.5	25 μL
Protein inhibitor cocktail	n/a	500 μL
ddH_2_O	n/a	48.325 mL
**Total**	**n/a**	**50 mL**
2. Before lysis the following compounds should be added:
  a) 25 μL DTT of a 1 M stock solution (final concentration 0.5 mM)
  b) 500 μL of a protein inhibitor cocktail (details are included in Key Resources Table)

### Enzymatic Assay Buffer

**Timing: 30 min**

3. Preparation of 1 L of sodium phosphate buffer (Table 2)
  a) 280 mL of a sodium phosphate mono salt solution (0.2 M)
  b) 720 mL of a sodium phosphate di salt solution (0.2 M)
  c) Recommended to check the final pH, which should be pH 7.2
  d) Buffer can be stored at 20°C for 3 months

**Table 2:** Recipe of Enzymatic Assay Buffer

Reagent	Final Concentration (mM)	Amount
Na_2_HPO_4_ (0.2 M)	100	720 mL
NaH_2_PO_4_ (0.2 M)	100	280 mL
**Total**	**n/a**	**1 L**
4. Before measurement of enzyme activity, prepare 20 mL of buffer for one 96-well plate and incubate at 37°C.
  a) Weigh out 1.5 mg NADPH and dissolve in 1 mL of assay buffer; after complete dissolution add the 1 mL to 19 mL assay buffer
  b) Add the specific substrate at a desired concentration to the buffer (e.g., for a maximum rate at substrate saturating conditions for methylglyoxal add 6 μL of a 40% solution; see Table 3 for detailed explanation)

**Table 3:** Possible Substrates and Appropriate Final Concentrations for Complete Michaelis-Menten Kinetics for Aldo-Keto Reductase

Substrate for AKR Activity	Final Concentration for Michaelis-Menten Kinetics (mM)	Amount to Add for 20 mL Assay Buffer (μL)
Methylglyoxal^a^ (1 M)	0.05; 0.1; 0.25; 0.5; 1; 2	1; 2; 5; 10; 20; 40
4-Hydroxynonenal^b^ (1 M)	0.1; 0.25; 0.5; 1; 2; 4	2; 5; 10; 20; 40
Acetaldheyde^b^ (1 M)	0.5; 1; 2.5; 5; 10; 20	10; 20;
DL-Glyceraldehyde (1 M)	0.1; 0.25; 0.5; 1; 2; 4	2; 5; 10; 20; 40
Malondialdehyde^c^ (1 M)	0.25; 0.5; 1; 2; 4; 8	5; 5; 10; 20; 40; 80

a Corrosive to metals, cause allergic skin reaction, serious eye irritation and is suspected of causing genetic defects. As a precaution, wear protective gloves. After contact with the skin or eyes, rinse cautiously the area with plenty of water for several minutes.

b Highly flammable and can cause eye irritation. Keep away from heat, hot surfaces, sparks, open flames and other ignition sources and do not smoke in the surroundings. If in contact with eyes, rinse cautiously with water for several minutes. Store in a well-ventilated place and keep container tightly closed.

c Can cause severe skin burns and eye damage. As a precaution, wear protective gloves, protective clothing, and eye and face protection. If in contact with eyes, rinse cautiously with water for several minutes. Immediately call a poison center/doctor if concerned.

**CRITICAL:** Many of the substrates (aldehydes, dicarbonyls etc.) for AKRs can be degraded and/or metabolized rapidly once they are diluted. It is therefore important to aliquot and freeze the desired concentrations of the specific substrate of interest. Substrates which are beyond the expiration date provided by the manufacturer should not be used. Always note the date of opening of the substrate in order to limit the degradation process.

## Key Resources Table

**Table:** 

REAGENT or RESOURCE	SOURCE	IDENTIFIER
**Chemicals, Peptides, and Recombinant Proteins**		

HEPES	Sigma-Aldrich	Cat# H3375
MgCl_2_	Sigma-Aldrich	Cat# M1028
KCl	Sigma-Aldrich	Cat# P9333
EDTA; ethylenediaminetetraacetic acid	Sigma-Aldrich	Cat# EDS
NP-40-alternative	Sigma-Aldrich	Cat# 492018
DTT; 1,4-dithiothreitol	Sigma-Aldrich	Cat# DTT-RO
Protease inhibitor cocktail	Sigma-Aldrich	Cat# P8340
NaH_2_PO_4_	Sigma-Aldrich	Cat# S9638
Na_2_HPO_4_	Sigma-Aldrich	Cat# S7907
NADPH	Sigma-Aldrich	Cat# NADPH-RO
MG; methylglyoxal	Sigma-Aldrich	Cat# M0252
GSH; L-Glutathione reduced	Sigma-Aldrich	Cat# G4251
β-Mercaptoethanol	Sigma-Aldrich	Cat# M6250
2-AAPA	Sigma-Aldrich	Cat# A4111
4-Hydroxynonenal	Sigma-Aldrich	Cat# 393204
Acetaldehyde	Sigma-Aldrich	Cat# 00070
DL-Glyceraldehyde	Sigma-Aldrich	Cat# G5001
Malondialdehyde	Sigma-Aldrich	Cat# 63287

**Software and Algorithms**		

MARS Data Analysis	BMG LABTECH	N/A
Reader Control	BMG LABTECH	N/A
GraphPad Prism 7	GraphPad Software	https://www.graphpad.com/scientific-software/prism/

**Other**		

FLUOstar OMEGA multiplate reader	BMG LABTECH	N/A
96-well plate, clear body, flat bottom	Thermo Scientific	Cat# 269620

## Materials and Equipment

In order to perform Michaelis-Menten kinetics and determine V_max_ and K_m_ for a specific substrate of AKR the concentration-ranges given in Table 1 should be used. It is recommended to calculate beforehand the appropriate volume of the stock solution for the substrate of interest. For each substrate concentration, it is necessary to prepare the individual amounts of assay buffer (e.g., for 6 substrates you will need to prepare 6 aliquots of assay buffer), to determine Michaelis-Menten kinetics.

## Step-By-Step Method Details

### Pulverization and Lysis of Tissue

**Timing: 2 h**

**Figure 1:**
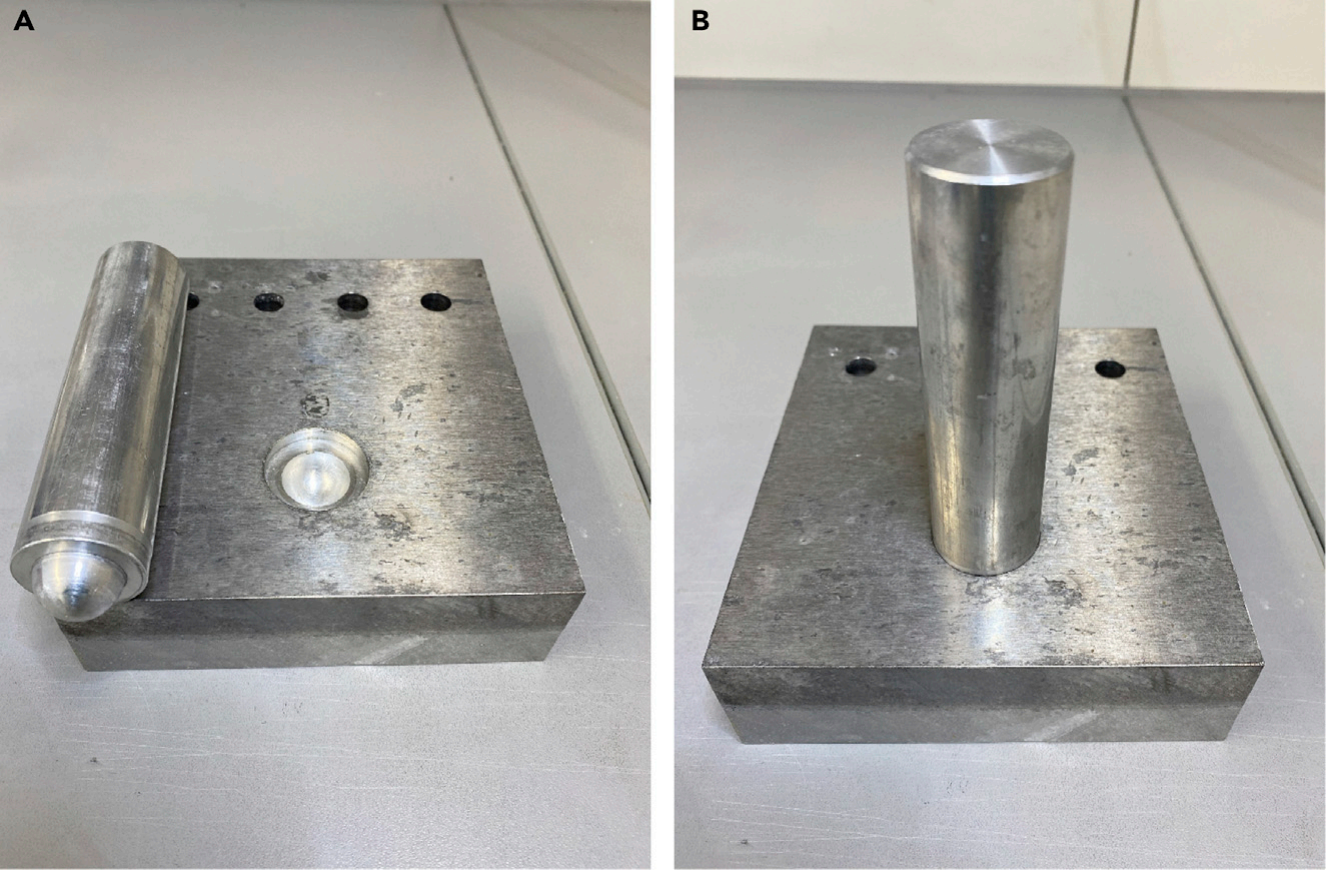
Self-Made Mortar and Pestle Construction for Grinding Frozen Tissue to Powder (A) Open. (B) Closed.

In order to maximize the yield of cytosolic proteins, the tissue of interest should be grounded to a fine powder prior to lysis. In our experience this is not achievable using electrical homogenizer, ultrasonic disruption techniques, or aggressive extraction buffers, which would interfere with the subsequent measurement of enzyme activity. Therefore, a common mortar and pestle is required. In our lab, we have designed a metal based mortar and pestle which allows for more efficient grinding of the tissues (Figures 1A and 1B and Methods Video S1). It is critical that the mortar and pestle as well as any additional tools such as spatulas are kept at an extremely low temperature in order to avoid thawing of the material (Troubleshooting 1). This is achievable using a bucket, which is filled permanently with liquid nitrogen (Methods Video S1). As it is difficult to effectively weigh the pulverized material without thawing, a reference to a filled "spoon" has been made in this protocol. For most samples, one "spoon" of tissue powder can then be used for 200 μL of lysis buffer. The lysis of the tissue has to be performed at 4°C. For every sample a new 1 mL syringe and 20-gauge needle should be filled with the appropriate amount of lysis buffer, depending upon the amount of samples and/or spoons taken (Methods Video S2).

1. Pulverization of tissue
  a) Place a tissue sample of around 0.3–10 mg in the precooled mortar
  b) Grind the tissue into a fine powder
    i. Take 1–5 "spoons" of powder and transfer it into 1.5 mL precooled tubes, which are then put on dry ice
    ii. Aspirate the remnants of powder with a vacuum pump in order to avoid contamination of the next tissue sample

**CRITICAL:** Every item which is in contact with the pulverized material should be precooled in liquid nitrogen. Including the mortar, pestle, tubes, spatulas, and whatever is needed when aliquoting the pulverized material. Experimenter should use 2–3 gloves to avoid cold burn when following this protocol.

2. Lysis of tissue
  a) For each "spoon" (~25 mg) of tissue, 200 μL of cytosolic lysis buffer is required.
  b) Add an appropriate amounts of lysis buffer to a syringe, ensuring that all air has been removed from the top of the needle
  c) Push the needle gently trough the lid of the tube
d) Homogenize the tissue by passing the lysis buffer 20 times through a 20-gauge needle (Methods Video S2)
e) Incubate the tissue homogenates on ice for 5 min and then centrifuge for 10 min at 10,500 × *g*. Protein concentration of the soluble fraction should be determined using the standard Bradford or BCA assay (Troubleshooting 2)
f) For enzyme activity measurement, samples should be diluted to final concentration of 5–10 μg/μL with lysis buffer

**CRITICAL:** During lysis with the needle and syringe, there is the potential for pressure to build up in the tube. This can be avoided by gently opening the lid of the tube every 5 times of buffer passing through the needle. It is also possible that the needle becomes blocked. If this occurs, then the needle should be replaced with a new one in order to avoid the increase of under-/overpressure in the tube.

Methods Video S1. Pulverization of Liver Tissue Using a Special Mortar and Pestle

Methods Video S2. Process of Lysis of Tissue Powder Using a Syringe Technique

### Aldo-Keto Reductase Enzyme Assay

**Timing: 2 h**

**Figure 2:**
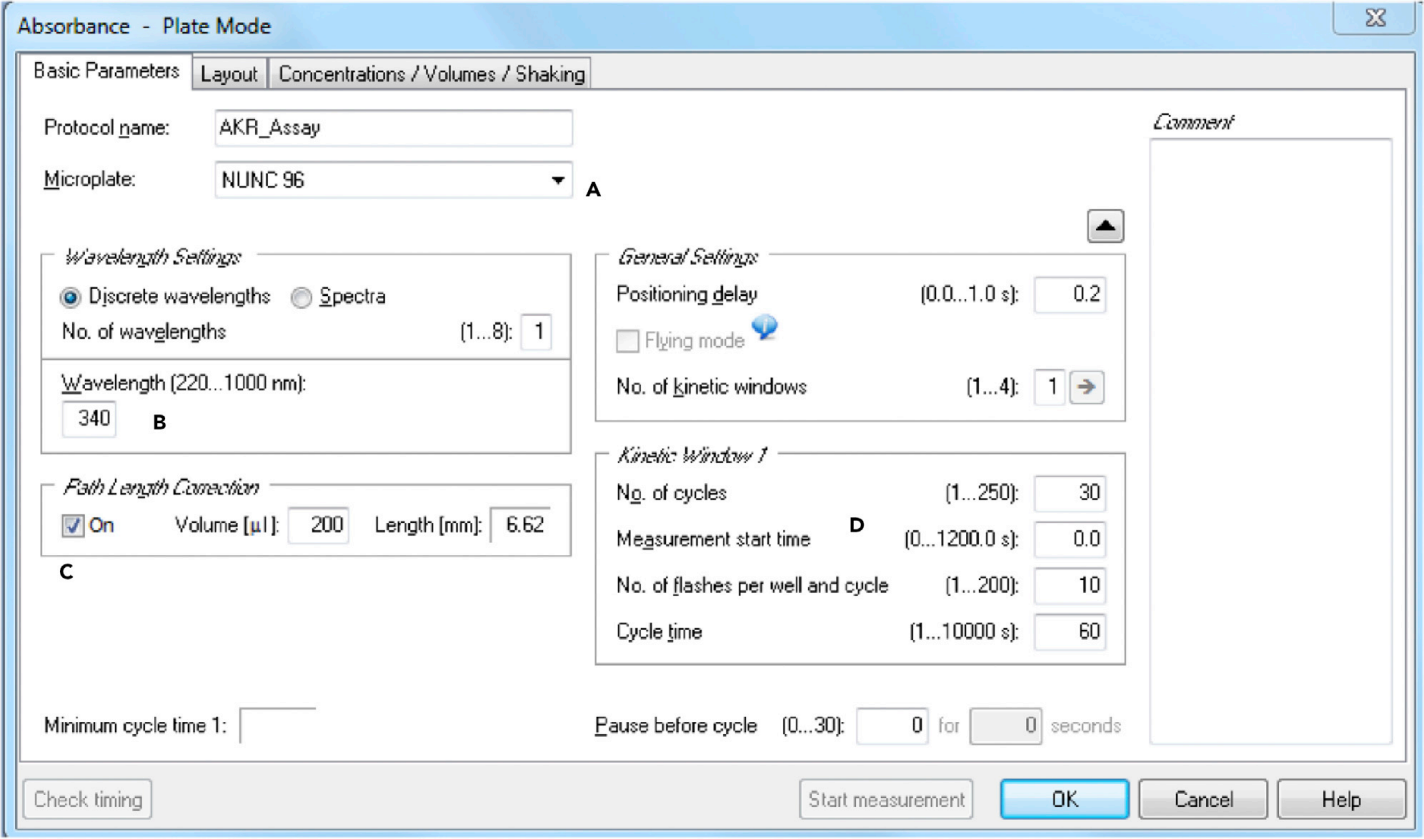
Software-Specific Settings at FLUOstar OMEGA Multiplate Reader (A) Microplate can be chosen on a drop down scale. (B) Wavelength has to be set to 340 nm. (C) Path length correction has to be turned on and depends on the microplate used (A) and the final volume in the plate. (D) One cycle per minute; 30 cycles in total should be recorded.

The enzyme activity of AKR is determined in 0.1 M sodium phosphate buffer (pH 7.2) containing the appropriate amount of substrate of interest (Table 1). The reaction is monitored at 37°C by following the loss of NADPH at 340 nm with a microplate reader. For each substrate concentration it is important to run four blanks in parallel in order to correct for autoxidation of NADPH. The setup at the microplate reader can differ between the manufacturers, but it is important to configure a path length correction which is defined by the used 96-well plate and the final volume (200 μL) (Figure 2).

3. Preparation of samples and blanks and plate loading
  a) For each sample and concentration prepare 125 μg of protein lysate in 25 μL assay buffer
  b) Blank 1 consists of enzyme assay buffer with the addition of 50 μL lysis buffer (substitutes the sample)
  c) A 96-well clear flat bottom microplate is loaded from top left to right bottom (Figure 3)

**Figure 3:**
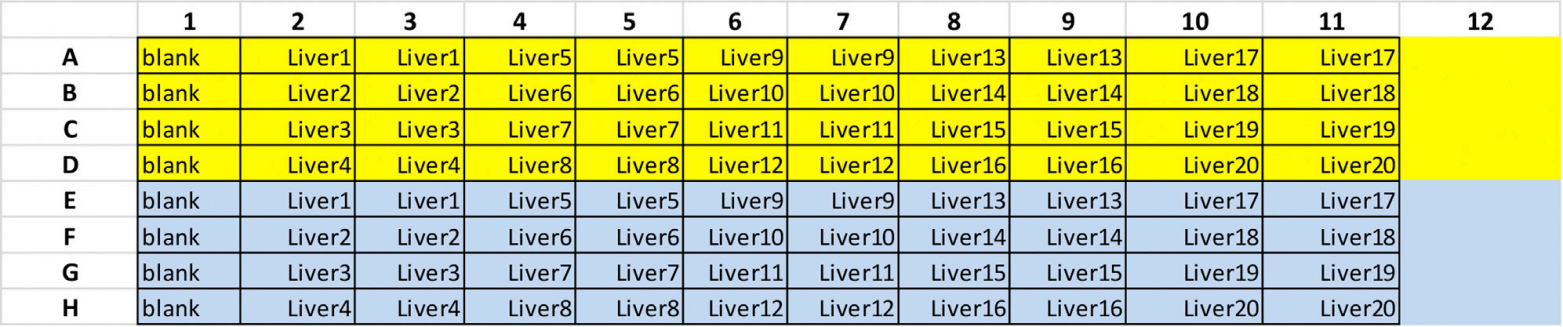
Possible Microplate Setup for 20 Liver Samples Quantifying Two Different Substrate Concentrations (Yellow/Turquoise)
  d) Blank should be run in quadruplicates and all samples are loaded in duplicates (10 μL per well)
  e) Finalize the assay buffer with the appropriate concentrations of the substrate of interest and NADPH
  f) Add 190 μL of the assay buffer in a prompt fashion using a multichannel pipette
4. Monitoring the loss of NADPH and calculation of enzyme activity
  a) Start to monitor the absorption at 340 nm over a period of 30 min with measurements every 1 min (Troubleshooting 3)
  b) After completion of the measurement period, the slope should be determined once it has a linear character
  c) Use the mean of the duplicate values to calculate the slope per minute and normalize the raw data of the slope/min by the blank
  d) The slope is normalized by the extinction of NADPH and protein content to achieve the U/mg (please refer to formula below) (Vander Jagt and Hunsaker, 2003)

$$\text{Enzyme activity }\left(\frac{\text{U}}{\text{mg}}\;\text{or}\frac{\frac{\text{μmol NADPH}}{\text{min}}}{\text{mg protein}}\right)=\;\frac{\text{normalized slope}/\text{min}}{-6.22}\times 20$$

***Optional:*** For some substrates in some tissues, it is possible to reduce the amount of cytosolic lysates (e.g., 20 μg/well) due to the naturally high basal enzyme activity of AKR (e.g., methylglyoxal in kidney tissue). The formula above has to be multiplied by 50 (instead of 20) in order to normalize for the lower total protein content. Furthermore, it is possible to run a positive control in your assay. This can be achieved by adding 0.01 μg/μL recombinant AKR1a1 or AKR1b1 protein, both representing the the isotypes with the highest activity and physiological relevance.

## Expected Outcomes

This protocol is useful in quantifying the kinetic parameters for AKRs driven detoxification parameters, such as V_max_ and K_m_ for different aldehyde substrates. It therefore displays the physiologically relevance of a tissue specific capacity to eliminate harmful compounds. Due to the large number of isotypes, the physiological relevance of AKRs cannot be obtained using mRNA or protein quantification, but only through the use of enzymatic assays which can demonstrate functionality (Mindnich and Penning, 2009).

## Quantification and Statistical Analysis

**Figure 4:**
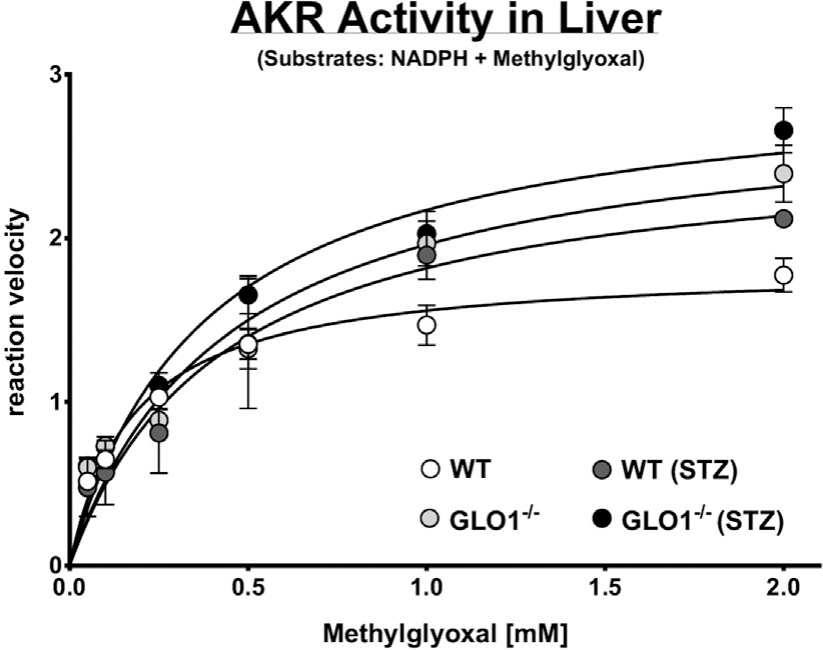
Michaelis-Menten Kinetic for Methylglyoxal in Liver Tissue of Four Different Mouse Model Systems WT, wild-type mouse; GLO1^−/−^, glyoxalase 1 knockout mouse; WT (STZ), wild-type mouse treated with streptozotocin; GLO1^−/−^ (STZ), glyoxalase 1 knockout mouse treated with streptozotocin. Each data point represents the mean ± SD of four individual liver samples from four animals. Adapted from Schumacher et al. (2018).

Using GraphPad (Prism) it is possible to fit (least square) a curve with nonlinear regression and subsequently calculate the kinetic parameters of a specific substrate and tissue (e.g., Figure 4).

Additional instructions for the calculation and how to use GraphPad can be found here: *https://www.graphpad.com/guides/prism/7/curve-fitting/reg_example_enzyme_kinetics.htm*.

## Limitations

This protocol quantifies total AKR driven catalysis of aldehydes of interest. It cannot, therefore, be used to distinguish the isotype specific capacity of a single AKR. It would be possible to determine isotype specific contribution to total AKR activity through the use of knockout models. However, studies suggest that majority of the detoxification efficiency of AKRs is dependent upon the isotypes AKR1a1 (also called aldehyde reductase) and AKR1b1 (also called aldose reductase)(Vander Jagt and Hunsaker, 2003). The protocol cannot be applied for adipose tissue due to high fat content in the lysates which disturbs the absorption measurement.

## Troubleshooting

### Problem 1

During tissue pulverization, the tissue starts to thaw, becoming sticky/clumpy and it cannot be aliquoted accordingly.

### Potential Solution

All tools should be put in liquid nitrogen at least 5 min before the processing of tissue begins. The mortar should be placed in a bucket filled with liquid nitrogen. Once the processing is finished, the tubes aliquoted with tissue powder should be kept on dry ice.

### Problem 2

The protein content of the lysates is too low to perform the assay.

### Potential Solution

If the amount of pulverized tissue is low, it is possible to reduce the volume of lysis buffer to a minimum of 50 μL. It is in general a good strategy to start with a lower volume of lysis buffer and dilute it, once the protein content has been determined.

### Problem 3

No detectable activity at 340 nm or the slope is positive instead of being negative as expected.

### Potential Solution

This can be the result of various mishandlings. Firstly, it is important to perform the tissue grinding, processing, and analysis on the same day. Once the protein lysates have been prepared and the protein content determined, the enzyme assay should be performed within 6 h. If there are any delays during lysis and enzyme assay, the lysates should be kept at 4°C and not frozen, as this will reduce the enzyme activity dramatically. Secondly, NADPH is very sensitive to minor changes in pH. Acidified or alkaline conditions can lead to autoxidation or aggregate formation of NADPH. Therefore, it is recommended to check the pH of the enzyme buffer. In some cases (e.g., high fat fed mouse models), the protein content of the lysates can precipitate in the well of the microplate and lead to turbidity. This will interact with the absorbance measurement making it impossible to monitor the decrease in NADPH.

## Resource Availability

### Lead Contact

Further information and requests for resources and reagents should be directed to and will be fulfilled by the Lead Contact, Jakob Morgenstern (jakob.morgenstern@med.uni-heidelberg.de).

### Materials Availability

The protocol did not generate any exclusive reagents, vectors, primers, or antibodies.

### Data and Code Availability

The protocol did not generate any exclusive data or code.
